# Transhiatal and transthoracic resection in adenocarcinoma of the esophagus: Does the operative approach have an influence on the long-term prognosis?

**DOI:** 10.1186/1477-7819-3-40

**Published:** 2005-06-24

**Authors:** Ines Gockel, Sina Heckhoff, Claudia M Messow, Werner Kneist, Theodor Junginger

**Affiliations:** 1 Department of General and Abdominal Surgery, Johannes Gutenberg-University, Mainz, Germany; 2 Institute of Biostatistics and Documentation, Johannes Gutenberg-University, Mainz, Germany

## Abstract

**Background:**

The goal of the present analysis was to investigate the long-term prognosis for adenocarcinoma of the esophagus treated with either the transhiatal (TH) or the transthoracic (TT) operative approach.

**Methods:**

Between September 1985 and March 2004, esophageal resection due to carcinoma was performed on a total of 424 patients. This manuscript takes into account the 150 patients suffering from adenocarcinoma of the esophagus in whom a transhiatal resection of the esophagus was performed. In the event of transmural tumor growth and a justifiable risk of surgery, the transthoracic resection was selected. An extended mediastinal lymph node dissection, however, was only carried out in the course of the transthoracic approach.

**Results:**

The transthoracic resection of the esophagus demonstrated a higher rate of general complications (p = 0.011) as well as a higher mortality rate (p = 0.011). The mediastinal dissection of the lymph nodes, however, revealed no prognostic influence. Considering all of the 150 patients with adenocarcinoma, as well as only those patients who had undergone curative resections (R0), the transhiatal approach was seen to demonstrate a better five-year survival rate of 32.1% versus 35.1%, with a median survival time of 24 versus 28 months, as compared with those who had undergone a transthoracic approach with a five-year survival rate of 13.6% (all patients) versus 17.7% (R0 resection) with a median survival time of 16 versus 17 months (p < 0.05).

**Conclusion:**

The prognosis in patients with adenocarcinoma of the esophagus is influenced by the depth of the tumor (pT) and the pM-category, as shown in the multivariate analysis. The present analysis did not demonstrate a relevant difference in survival for patients with N0 and N1 stages undergoing transhiatal or transthoracic esophagectomy. It is questionable, if a more extensive mediastinal lymph node dissection, in addition to the clearance of abdominal lymph nodes, offers prognostic advantages in adenocarcinoma of the esophagus. However, the morbidity and mortality associated with the transthoracic approach is higher.

## Background

The adequate operative procedure for adenocarcinoma of the esophagus is currently being discussed with a great deal of controversy. The question is whether or not the prognosis can be improved through an extended lymph node dissection by the transthoracic approach as compared with the use of the transhiatal procedure combined with removal of the posterior, lower mediastinal lymph nodes. A randomized study comparing both operative procedures revealed a higher morbidity following the transthoracic approach, but no significant improvement in the prognosis even when including tumors of the esophagogastric junction [1]. Our own approach includes the transhiatal resection as a routine procedure and demonstrates a preference for the transthoracic approach in the event of a suspected tumor infiltration of intrathoracic or of an affliction of the mediastinal lymph nodes.

An analysis of 150 consecutive patients with adenocarcinoma of the esophagus, who had undergone esophageal resection and were evaluated prospectively, was carried out in order to investigate the significance of the surgical procedure on the prognosis.

## Patients and methods

In 424 prospectively-evaluated patients, esophageal resections had been performed between September 1985 and March 2004 due to the existence of malignant tumors. The current data analysis, however, involves 150 patients with histologically verified adenocarcinoma of the esophagus, of whom 135 were Barrett's carcinoma. Adenocarcinoma enrolled in this study only included Siewert type I tumors. Type II (tumors of the cardia) and type III (subcardial tumors with infiltration of the cardia) were strictly not taken into consideration.

For preoperative staging, EUS (endoscopic ultrasound), CT (computed tomography) of the neck, chest and abdomen and PET (positron emission tomography) were routinely carried out.

Aside from the selected surgical procedure, the following parameters were documented:

The duration of surgery (incision-to-suture time), intraoperative blood loss as well as the intraoperative substitution of blood, time spent in the intensive care unit, tumor staging according to the UICC classification [2], number of tumor-free and tumor-infiltrated abdominal as well as mediastinal lymph nodes, surgical complications (anastomotic insufficiency, transplant necrosis, postoperative bleeding, tracheal fistula, recurrent laryngeal nerve paralysis, chylothorax), general complications (pneumonia, ARDS = adult respiratory distress syndrome, pulmonary embolism, decompensated cardiac insufficiency, myocardial infarction, transitional syndrome, renal insufficiency), duration of the postoperative hospital stay, mortality (30-day and hospital mortality rate) as well as the survival time have all been recorded. Until April 30, 2004, the end of the observation period for this study, the long-term follow-up was recorded and evaluated. It was possible to follow-up the post-stationary course of development in each of these 150 patients.

For adenocarcinoma of the esophagus, a transhiatal resection of the esophagus was carried out as a routine procedure. Patients with transmural tumor spread and suspected intrathoracic lymph nodes involvement and a justifiable risk of surgery, underwent a surgical procedure employing the transthoracic approach. All together, 103 patients underwent transhiatal and 47 patients transthoracic resection. The transthoracic surgical approach was performed via a right dorso-lateral thoracotomy, and the esophageal substitute consisted of a gastric pull-up located in the anatomical esophageal bed prevertebrally. During transthoracic esophageal resection, the anastomosis was usually done intrathoracically with a stapler (EEA 25 mm with an additional suture line by hand), whereas patients undergoing the transhiatal procedure had a cervical, hand-sewn anastomosis (end-to-side esophagogastrostomy with two suture lines).

The transhiatal procedure was carried out with an abdominal lymph node dissection (including the paracardial nodes, the left gastric artery nodes along with the lymph nodes of the lesser curvature of the stomach, the celiac trunk, the common hepatic artery and in selected cases – as macroscopic tumor involvement – the splenic artery), as well as with an excision of the lymph nodes extending as far as the carina of the trachea, and to those lymph nodes which could be reached in the lower, posterior mediastinum. The transthoracic technique involved an abdominal (as described above) and a more extensive mediastinal lymphadenectomy in the sense of a two-field dissection. The specimen here included the lower and middle mediastinal, subcarinal, and right-sided paratracheal lymph nodes (*en bloc *dissection). Paratracheal and bifurcal nodes were only removed on both sides in case of clinical suspicion of bilateral involvement. The aortopulmonary – window nodes were dissected separately. About 90% of the patients with adenocarcinomas (n = 133) demonstrated a tumor localization in the distal third of the esophagus.

### Statistical analysis

In order to make a statistically-evaluative comparison between the operative procedures, a minimum of two, non-parametric statistical tests were applied to the measured parameters, making use of cross-tabulation analyses (in association with Fisher's exact test), which were employed for all categorized parameters involving (a) parameters that are considered to be nominal variables or (b) variables with a specific, ordinal scale value demonstrating only *two *intensities or categories.

Additionally, the Mann-Whitney U test (M-W test or U-test) was used for all of the parameters which could be classified according to an ordinal scale and which also demonstrated *more than two *intensities or categories.

The life-table and death-table analyses were primarily performed in order to calculate the three-and five-year survival rate for subgroups, following either one of the two surgical procedures. The median survival time, the specific survival curves of the various random samples, were determined by Kaplan-Meier analyses (K-M analyses). Differences in survival between the groups were assessed with the log-rank test. Multivariate survival analysis was performed using the Cox proportional hazard model. As no adjustment for multiple testing was performed, p-values should be considered as descriptive.

Median, minimum and maximum values, as well as the arithmetic mean and standard deviation, were also collected for the descriptive statistics, accompanied by the respective number of valid cases.

## Results

With regard to age, ASA classification, tumor localization, metastasis as well as tumor grading, there was no statistically significant difference between patients undergoing the transhiatal or the transthoracic approach.

For those patients who had undergone transhiatal resections, individuals demonstrating both pT-category 1 and 2 were seen to be more frequent, compared to pT3-category. These patients also had fewer lymph node metastases (59% versus 78.3%, p = 0.027). Consequently, UICC stages I and II were seen to be more prevalent in those patients who had undergone transhiatal procedures (p < 0.0005) (Table 1). Tumor-free margins proximal, distal as well as circumferential (R0 resections) were observed more frequently following a transhiatal resection (92.2%) as compared with those treated using a transthoracic approach (78.7%) (p = 0.029) (Table 1).

**Table 1:** Clinicopathological features

**Clinicopathological features**										
	**total**			**transhiatal (TH)**			**transthoracic (TT)**			**p-value**
	**(n = 150)**			**(n = 103)**			**(n = 47)**			

**age (years)**										
*median (range)*	61 (28–78)			63 (28–78)			60 (35–75)			0.199
U-test										
**ASA classification**										
*ASA II*	62	145	42.8%	44	99	44.4%	18	46	39.1%	0.592
*ASA III-IV*	83	145	57.2%	55	99	55.6%	28	46	60.9%	
Fisher's exact test										
**R classification**										
*RO*	131	149	87.9%	94	102	92.2%	37	47	78.7%	0.029
*R1, R2*	18	149	12.1%	8	102	7.8%	10	47	21.3%	
Fisher's exact test										
**tumor localization**										
*upper third*	1	149	0.7%	0	103	0.0%	1	46	2.2%	
*middle third*	15	149	10.1%	8	103	7.8%	7	46	15.2%	0.155
*lower third*	133	149	89.3%	95	103	92.2%	38	46	82.6%	
Chi-Square (Pearson)										
**pT category**										
*pT1*	28	150	18.7%	27	103	26.2%	1	47	2.1%	
*pT2*	39	150	26.0%	34	103	33.0%	5	47	10.6%	<0.0005
*pT3*	77	150	51.3%	41	103	39.8%	36	47	76.6%	
*pT4*	6	150	4.0%	1	103	0.9%	5	47	10.6%	
Chi-Square (Pearson)										
**pN category**										
*pN0*	51	146	34.9%	41	100	41.0%	10	46	21.7%	0.027
*pN1-2*	95	146	65.1%	59	100	59.0%	36	46	78.3%	
Fisher's exact test										
**pM category**										
*pM0*	105	149	70.5%	75	102	73.5%	30	47	63.8%	0.250
*pM1*	44	149	29.5%	27	102	26.5%	17	47	36.2%	
Fisher's exact test										
**UICC stage**										
*UICC I-II*	62	149	41.6%	53	102	52.0%	9	47	19.2%	<0.0005
*UICC III-IV*	87	149	58.4%	49	102	48.0%	38	47	80.9%	
Chi-Square (Pearson)										
**tumor grading**										
*G1*	10	146	6.9%	8	100	8.0%	2	46	4.4%	0.167
*G2*	43	146	29.5%	32	100	32.0%	11	46	23.9%	
*G3*	76	146	52.1%	46	100	46.0%	30	46	65.2%	
*G4*	17	146	11.6%	14	100	14.0%	3	46	6.5%	
Chi-Square (Pearson)										
**tumor length **(grouped)										
*0–4 cm*	69	143	48.3%	52	98	53.1%	17	45	37.8%	0.225
*>4–8 cm*	62	143	43.4%	39	98	39.8%	23	45	51.1%	
*>8 cm*	12	143	8.4%	7	98	7.1%	5	45	11.1%	
Chi-Square (Pearson)										

### Morbidity and mortality

The median time taken duration surgery for patients undergoing transhiatal resection was 254 minutes as compared with 335 minutes (without including the time required to change the position of the patient) for those undergoing a transthoracic resection (p < 0.0005).

The intraoperative blood loss was seen to be 800 ml (under the transhiatal procedure) in contrast to 1000 ml (using the transthoracic approach) (p = 0.002). The intraoperative blood substitution was a median of 0 units of packed red blood cells (PRBC) in the course of the transhiatal versus 1 unit during the transthoracic resection (p = 0.128) (Table 2).

**Table 2:** Morbidity and mortality

		**transhiatal (TH)**			**transthoracic (TT)**			**p-value**
**duration of surgery**	median (range)	254	(160–485)		335	(240–540)		<0.0005
(min)								U-test
**blood loss**	median (range)	800	(0–7500)		1000	(200–5000)		0.002
(ml) intraoperatively								U-test
**blood substitution**	median (range)	0	(0–6)		1	(0–14)		0.128
(PRBC) intraoperatively								U-test
**surgical complications**		44	103	**42.7%**	7	47	**14.9%**	0.001
								Fisher
**general complications**		22	102	**21.6%**	20	47	**42.6%**	0.011
								Fisher
**total complications**		62	103	**60.2%**	25	47	**53.2%**	0.477
								Fisher
**duration of postoperative intensive care unit stay**								
(days)	median (range)	8	(2–107)		8	(1–97)		0.566
								U-test
**duration of postoperative in-hospital stay**								
(days)	median (range)	21	(14–126)		20	(13–107)		0.646
**hospital mortality**		3	103	**2.9%**	7	47	**14.9%**	0.011
								Fisher
								
**30-day mortality**		2	100	**2.0%**	7	47	**14.9%**	0.005
								Fisher

The rate of surgical complications, with 42.7% (44/103) for the transhiatal approach, was higher than that observed in the course of a transthoracic resection (14.9%) (7/47) (p = 0.001), primarily as a result of an insufficiency of the cervical anastomosis, which could nevertheless be treated conservatively in all cases. The rate of general complications, with 42.6% (20/47) versus 21.6% (22/102) (p = 0.011), was higher following the transthoracic approach, which was associated with a higher rate of pneumonia. The median duration spent in the intensive care unit (8 days after both procedures; p = 0.566), as well as the median postoperative in-hospital stay (21 versus 20 days; p = 0.646), did not differ between the two types of resection (Table 2).

The hospital mortality for the entire population of patients was seen to be 6.7% (10/150). Following the transhiatal procedure, the 30-day (p = 0.005) and the hospital mortality (p = 0.011) were lower than that observed following the transthoracic approach (2.0 and 2.9% versus 14.9%) (Table 2). Causes of death were related to pulmonary sepsis in one patient of the TT group only (the others were due to cardiovascular problems (n = 5) and anastomotic insufficiency with sepsis (n = 1)). Causes of deaths following the TH-resection were pulmonary sepsis (n = 1), hemorrhagic shock (n = 1) and acute rupture of the mitral valve tendon (n = 1).

### Lymph node status

The total number of lymph nodes dissected in the course of transhiatal resection were lower (19; range 0–55) than that observed during the transthoracic procedure (31, range 2–94) (p < 0.0005) (Table 3). The number of afflicted mediastinal lymph nodes dissected in the course of transhiatal resection, correspondingly, was lower as well (2 versus 4; p = 0.030). The lower number of excised lymph nodes could be based on the lower number of mediastinal lymph nodes which were seen to have been removed in the course of the transhiatal approach (3 versus 9, p < 0.0005) (Table 3). Correspondingly, the number of afflicted, mediastinal lymph nodes dissected during transhiatal resection was lower (0 versus 2; p < 0.0005) (Table 3). The number of abdominal lymph nodes which were excised was higher under the transhiatal in contrast to the transthoracic approach (14 versus 11, p = 0.017). The number of afflicted, abdominal lymph nodes, however, revealed no difference between the two operative procedures. The median lymph node-ratio (quotient between the number of tumor-infiltrated lymph nodes and the total number of lymph nodes dissected) during transthoracic resection was seen to be 0.10 versus 0 for the transhiatal as compared with 0.154 versus 0.111 for the transthoracic procedure, respectively (p > 0.05) (Table 3).

**Table 3:** Lymph node (LN) dissection

	**transhiatal (TH)**			**transthoracic (TT)**			
	median	range	mean	median	range	mean	U-test
total LN dissected	19	(0–55)	21.3	31	(2–94)	32.4	<0.0005
positive LN dissected	2	(0–33)	5.4	4	(0–76)	7.9	0.030
							
total abdominal LN	14	(0–48)	16.4	11	(0–51)	12.0	0.017
positive abdominal LN	1	(0–28)	3.4	1	(0–17)	3.1	0.802
							
total thoracic LN	3	(0–42)	5.1	19	(2–83)	20.2	<0.0005
positive thoracic LN	0	(0–30)	1.5	2	(0–67)	4.8	<0.0005
							
LN ratio total	0.11	(0–0.86)	0.20	0.17	(0–0.81)	0.24	0.160
LK ratio abdominal	0.10	(0–0.89)	0.17	0.15	(0–1)	0.25	0.197
LK ratio thoracic	0.00	(0–1)	0.22	0.11	(0–1)	0.24	0.156

### Prognosis

**Figure 1:**
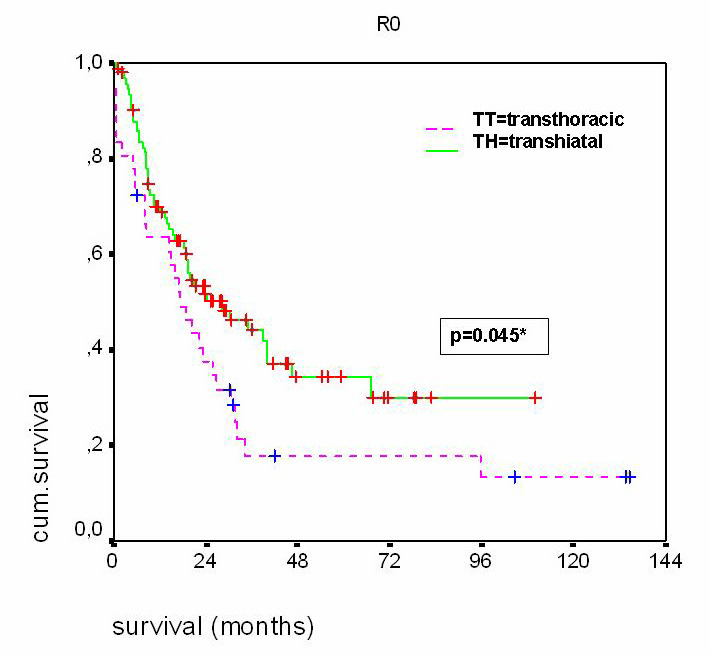
Kaplan-Meier survival curves for patients with adenocarcinoma and R0 resection undergoing transhiatal (TH) and transthoracic (TT) esophagectomy.

**Figure 2:**
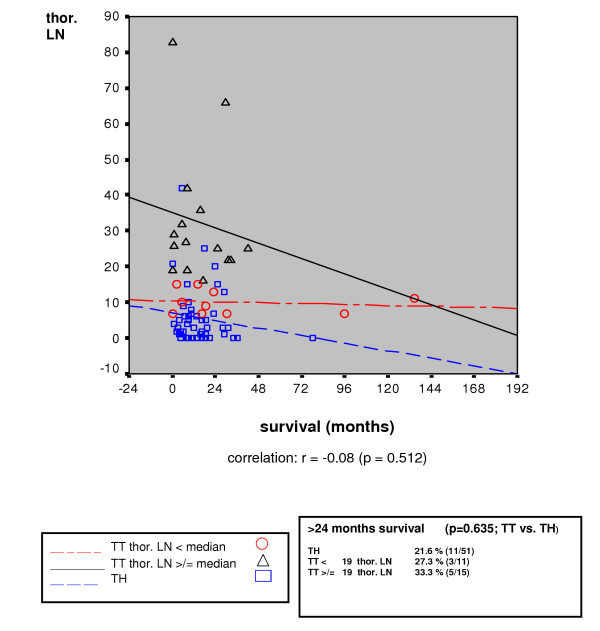
Correlation between long-term survival and the number of dissected thoracic lymph nodes (LN) for curative (R0) resected, nodal positive (pN1) patients (n = 77) with adenocarcinoma of the esophagus.

The median and the five-year survival rates for the total patient population were markedly better following transhiatal resection than subsequent to the transthoracic procedure (16 months and 32.1% as compared with 16 months and 13.6%, respectively; p = 0.018) (Table 4). For patients with curative (R0) resection, the prognosis was also seen to be better for those patients who underwent transhiatal esophagectomy (28 months versus 17 months; p = 0.045) (Figure 1). In patients without lymph node metastases (pN0), the median survival time following an R0 resection and a transhiatal approach was 67 months (n = 40) as compared to 27 months for those who had undergone transthoracic esophagectomy (n = 9) (p > 0.05). For the patients who were seen to be nodal-positive (pN1), those who had undergone a transhiatal resection (n = 51) demonstrated a median survival time of 16 months contrasted to 14 months for those treated using a transthoracic approach (n = 26) (p > 0.05). An expansion in the number of mediastinal lymph nodes which had been dissected to include more than 19 lymph nodes (=median), however, was not seen to have any positive effect in this group (Table 4). In the scatter diagram (Figure 2), there was no correlation seen between the long-term prognosis of adenocarcinomas of the esophagus and the number of dissected mediastinal lymph nodes.

**Table 4:** Prognosis

	**operative approach**	**n**	**median survival (months)**	**5-year survival rate**	**p-value**
		
total	TT	46	16	13.6%	0.018
	TH	103	24	32.1%	
curative (R0)	TT	36	17	17.6%	0.045
	TH	94	28	35.1%	
palliative (R1)	TT	10	9	-	0.778
	TH	8	8	-	
R0 pN0	TT	9	27	22.2%	0.072
	TH	40	67	54.1%	
R0 pN1	TT	26	14	14.3%	0.430 a
	<19 thor. LN	12	17	16.7%	0.829 b
	>/ = 19 thor. LN	14	8	-	
	TH	51	16	22,4%	0.703 c
R0 pN1 T1-2	TT	4	14	25.0%	0.357
	TH	21	28	33.3%	
R0 pN1 T3-4	TT	22	9	12.2%	0.793
	TH	30	10	-	
R0 pN1 ASA 1–2	TT	11	16	20.0%	0.345
	TH	23	19	33.3%	
R0pN1 ASA 3–4	TT	15	9	10.3%	0.999
	TH	25	10	-	
R0 middle third	TT	4	5	25%	0.931
(tumor localization)	TH	8	24	15%	
R0 lower third	TT	32	17	17.1%	0.044
(tumor localization)	TH	86	30	37.9%	

TT: Transthoracic, TH: Transhiatal

a = (TT vs. TH)

b= (TT intern)

c = (TT thor. LN vs. TH)

A relevant advantage to the transhiatal in comparison with the transthoracic operative procedure (n = 32; median survival time: 17 months; p = 0.044) was also to be seen for tumor localizations in the distal third of the esophagus (n = 86; median survival time: 30 months). For the minority of patients with a tumor localization in the middle third, however, there was no significant difference between these two surgical approaches (TT : 4, TH : 8).

In the multivariate survival analysis, the simultaneous influence of the surgical approach, R-classification, pT-, pN-, pM-category, tumor grading, ASA classification, and age was assessed. Only the pM-(p = 0.020) and pT-category (p = 0.009) had a relevant influence on survival (Table 5). In a forward variable selection pT and pM were the only variables that were selected.

**Table 5:** Multivariate analysis of prognostic factors for survival

		**95%-confidence interval**		
**variable**	**hazard ratio**	**lower limit**	**upper limit**	**p-value**

**R-classification**				
-R1 and R2 vs. R0	1.368	0.772	0.2424	0.284

**pT-category**				**0.009**
-pT2 vs. pT1	2.418	0.952	6.144	0.063
-pT3 and pT4 vs. pT1	3.930	1.569	9.847	0.003

**pN-category**				
-pN1, 2 vs. pN0	1.398	0.776	2.526	0.267

**tumor grading**				**0.659**
G2 vs. G1	0.731	0.264	2.021	0.545
G3 vs. G1	0.993	0.370	2.666	0.989
G4 vs. G1	1.082	0.348	3.360	0.892

**ASA-classification**				**0.777**
ASA III vs. ASA II	1.100	0.680	1.779	0.699
ASA IV vs. ASA II	1.391	0.544	3.558	0.491

**pM-category**	1.782	1.096	2.898	**0.020**

**surgical approach**				
TT vs. TH	0.937	0.592	1.482	0.781

**age**	0.997	0.972	1.023	**0.839**

5-year survival rates for **R0**-resections according to the different UICC stages for the whole group were: stage I (n = 20): 56.3%; stage IIa (n = 20): 26.5%; stage IIb (n = 16): 16.5%; stage III (n = 34): 34.4%; and stage IV (n = 31): 14.8%. Multivariate analysis of three-and five-year survival including surgical approach and UICC staging showed no significant influence of the surgical approach, neither with respect to the three-nor to the five-year survival (3 years: p = 0.289; 5 years: p = 0.685) (Table 6, 7).

**Table 6:** Multivariate analysis of three-year survival

		**95%-confidence interval**		
**variable**	**odds ratio**	**lower limit**	**upper limit**	**p-value**

**surgical approach**				
TT vs. TH	1.931	0.573	6.514	0.289

**UICC**				**0.001**
-IIa vs. I	1.866	0.459	7.583	0.383
-IIb vs. I	20.350	2.132	194.254	0.009
-III vs. I	5.967	1.400	25.439	0.016
-IV vs. I	46.422	5.071	424.994	0.001

Odds ratios are to be interpreted as odds for death so that they are interpretable in the same way as the hazard ratios in Table 5.

**Table 7:** Multivariate analysis of five-year survival

		**95%-confidence interval**		
**variable**	**odds ratio**	**lower limit**	**upper limit**	**p-value**

**surgical approach**				
TT vs. TH	0.716	0.143	3.594	0.685

**UICC**				**0.020**
-IIa vs. I	5.483	0.796	37.788	0.084
-IIb vs. I	8.575	0.850	86.488	0.068
-III vs. I	12.133	1.626	90.526	0.015
-IV vs. I	28.124	2.773	285.211	0.005

Odds ratios are to be interpreted as odds for death so that they are interpretable in the same way as the hazard ratios in Table 5.

## Discussion

The present investigation is based on 150 consecutive patients who had undergone surgery for adenocarcinoma of the esophagus and whose peri-and postoperative course of was evaluated prospectively. The transhiatal approach with the posterior excision of the lower mediastinal lymph nodes was employed as a routine procedure. In the event that a tumor was to be found in the vicinity of the tracheal bifurcation, or should there be suspicion of an infiltration of intrathoracic organs or mediastinal lymph node metastases, a transthoracic approach with two-field lymphadenectomy was selected.

The study verified the varying perioperative risks of the transthoracic and transhiatal procedures. With the same age distribution and the same risk factors, measured according to the ASA classification, the 30-day and hospital mortality following the transhiatal approach were seen to be markedly reduced as compared with the transthoracic procedure. This operative technique, however, primarily led to general complications, especially of pulmonary kind, which served to bring about an unfavorable course of development. The relatively high mortality rate after the transthoracic procedure reflects patients seen during the whole study period including the earlier interval. Recently, probably due to improved surgical techniques and advanced intensive care therapy, the rate was seen to be much lower compared to the first study period. Following transhiatal esophagectomy, the surgical complications predominated, especially the insufficiency of the cervical esophagogastrostomy, a condition which was nevertheless seen to close in all cases subsequent to a conservative therapy. The reduced general risk of the transhiatal procedure has been confirmed in most of the available studies [3-6]. On the other hand, though exerting a higher operative risk, transthoracic resection intends to improve long-term survival by wide excision of the tumor and peritumoral tissue with extended en bloc mediastinal lymphadenectomy [7-11].

The influence of the operative procedure on the prognosis in adenocarcinoma of the esophagus has been investigated in a number of retrospective and in one prospective, randomized study [1]. The disadvantage of the blunt transhiatal resection is the reduced transthoracic lymph node dissection, which is unfortunately limited to the lower posterior, mediastinal lymph nodes [12-15]. In the present investigation, a higher number of mediastinal lymph nodes were consequently seen to be excised in the course of transthoracic resections, a situation which has also resulted in an increase in the number of involved lymph nodes. The number of afflicted, abdominal lymph nodes was seen to be similar following both of the two types of operative procedures.

Our data show that through transhiatal esophagectomy, a five-year survival rate of 54.1% was seen in patients without (pN0) and of 22.4% in those with lymph node involvement (pN1). Patients undergoing transthoracic resection without afflicted lymph nodes demonstrated no improvement in prognosis (22.2%) as a result of this procedure, although only a smaller number of such cases were observed here. Findings derived from studies investigating the spread of lymph nodes in cases of adenocarcinoma allow one to conclude that the lymph node metastases associated with distal adenocarcinomas are initially seen to metastasize into the lymph nodes in the vicinity of the tumor and only later into the lymph nodes of the upper mediastinal region [16]. A counter argument is a likelihood of cervical lymph node involvement as high as 3.5% reported for T3 adenocarcinoma of the distal esophagus and the gastro-esophageal junction [17]. Thus, the transhiatal procedure must consequently be considered to be adequate at least for patients without any lymph node involvement.

In cases with lymph node involvement, the prognosis of our own patient population was independent of the surgical procedure and, consequently, also of the extent of lymph node dissection. The transhiatal esophagectomy, because of the reduced morbidity and mortality and the presence of more early stage tumors with correspondingly more frequent R0 resections associated with this approach, however, was seen to result in a better long-term prognosis. Thus, the present study was not randomized and the survival benefit seen after transhiatal esophagectomy might therefore be based on a more favorable patient selection. This needs to be checked in a randomized controlled trial.

The higher number of advanced stages in the transthoracic group is certainly explained by the marked difference in the pT-category (p < 0.0005) and also possibly by stage migration due to the more extended lymph node dissection (pN-category: p = 0.027) (Table 1).

In the event of existing lymph node metastases, this indicates that a generalized disease is present which can no longer be influenced by local, surgical measures. In the multivariate analysis of potential factors with an influence on the long-term survival rate of our patient population, the pT-and pM-category were seen to represent independent, prognostic parameters. In accordance with this, the findings of other authors must also be expounded upon, which demonstrated, following extensive lymph node dissection in cases of adenocarcinoma, that the most favorable results were to be observed in the event of lacking or only minimal lymph node involvement [1].

The question of whether or not a neoadjuvant (radio-) chemotherapy can lead to an improvement in the prognosis, as verified in one study, but not confirmed in another, cannot presently be answered conclusively [18,19].

The prognosis following an R1 resection, subsequent to both – transthoracic as well as transhiatal – approaches, with survival times of 8 or 9 months, respectively, must be considered unfavorable, so that this situation must be avoided whenever possible.

For the operative procedure involving distal adenocarcinomas of the esophagus, the present results permit one to make the following conclusions: The transhiatal procedure together with a posterior, lower mediastinal lymph node dissection is associated with a comparably reduced perioperative risk and, for patients in whom a radical tumor excision is possible, represents an oncologically adequate method. The prognosis following R0 resection is defined by the T-and N-categories. In the event of an N1 situation, the prognosis must be considered unfavorable, independent of the operative procedure selected. According to the present investigation including a lack of randomization, it is questionable, if the long-term survival can be improved through the expansion of a lymph node dissection using the transthoracic procedure. This approach, however, can then be indicated when a complete resection of the tumor is not to be achieved by transhiatal esophagectomy, for instance when the tumor is seen to demonstrate a close relationship to the tracheal bifurcation, the tumor is suspected to involve an infiltration of intrathoracic structures or in the event of suspected lymph node metastases in the upper mediastinal region.

## Conclusion

The present analysis did not demonstrate a relevant difference in survival for patients with N0 and N1 stages undergoing transhiatal or transthoracic esophagectomy. It is questionable, if an extensive mediastinal lymph node dissection in addition to the clearance of abdominal lymph nodes offers any prognostic advantages in adenocarcinoma of the esophagus also probably due to the increased morbidity and mortality associated with the transthoracic approach.

## Competing interests

The author(s) declare that they have no competing interests.

## Authors' contributions

**IG: **study design, collection of data, statistical analysis, sequence alignment, draft of manuscript

**SH: **collection of data, statistical analysis

**CMM: **statistical analysis

**WK: **study design, collection of data, statistical analysis

**ThJ: **conceived of the study, design and coordination of the study, draft and revision of the manuscript

## References

[B1] Hulscher JB, van Sandick JW, de Boer AG, Wijnhoven BP, Tijssen JG, Fockens P, Stalmeier PF, Ten Kate FJ, van Dekken H, Obertop H, Tilanus HW, van Lanschot JJ (2002). Extended transthoracic resection compared with limited transhiatal resection for adenocarcinoma of the esophagus. N Engl J Med.

[B2] Wittekind CH, Wagner G (1997). TNM-Klassifikation maligner Tumoren.

[B3] Orringer MB, Marshall B, Stirling MC (1993). Transhiatal esophagectomy for benign and malignant disease. J Thorac Cardiovasc Surg.

[B4] Pac M, Basoglu A, Kocak H, Yekeler I, Yediyildiz S, Aydin NE, Yilm A, Okcu N, Keles M (1993). Transhiatal versus transthoracic esophagectomy for esophageal cancer. J Thorac Cardiovasc Surg.

[B5] Fok M, Siu KF, Wong J (1989). A comparison of transhiatal and transthoracic resection for carcinoma of the thoracic esophagus. Am J Surg.

[B6] Boyle MJ, Franceschi D, Livingstone AS (1999). Transhiatal versus transthoracic esophagectomy: complication and survival rates. Am Surg.

[B7] Müller JM, Erasmi H, Stelzner M, Zieren U, Pichlmaier H (1990). Surgical therapy of esophageal carcinoma. Br J Surg.

[B8] Chu KM, Law SY, Fok M, Wong J (1997). A prospective randomized comparison of transhiatal and transthoracic resection for lower-third esophageal carcinoma. Am J Surg.

[B9] Goldminc M, Maddern G, Le Prise E, Meunier B, Campion JP, Launois B (1993). Oesophagectomy by transhiatal approach or thoracotomy: a prospective randomized trial. Br J Surg.

[B10] Jacobi CA, Zieren HU, Müller JM, Pichlmaier H (1997). Surgical therapy of esophageal carcinoma: the influence of surgical approach and esophageal resection on cardiopulmonary function. Eur J Cardiothorac Surg.

[B11] Hulscher JBF, Tijssen JGP, Obertop H, van Lanschot JJB (2001). Transthoracic versus transhiatal resection for carcinoma of the esophagus: a meta-analysis. Ann Thorac Surg.

[B12] Akiyama H, Tsurumaru M, Udagawa H, Kajiyama Y (1994). Radical lymph node dissection for cancer of the thoracic esophagus. Ann Surg.

[B13] Roder JD, Busch R, Stein HJ, Fink U, Siewert JR (1994). Ratio of invaded to removed lymph nodes as a predictor of survival in squamous cell carcinoma of the oesophagus. Br J Surg.

[B14] Siewert JR, Stein HJ (1999). Lymph node dissection in squamous cell esophageal cancer – who benefits?. Langenbecks Arch Surg.

[B15] Tsurumaru M, Kajiyama Y, Udagawa H, Akiyama H (2001). Outcomes of extended lymph node dissection for squamous cell carcinoma of the thoracic esophagus. Ann Thorac Cardiovasc Surg.

[B16] Tachimori Y, Kato H, Watanabe H, Sasako M, Kinoshita T, Maruyama K (1996). Difference between carcinoma of the lower esophagus and the cardia. World J Surg.

[B17] Van de Ven C, De Leyn P, Coosemans W, Van Raemdonck D, Lerut T (1999). Three-field lymphadenectomy and pattern of lymph node spread in T3 adenocarcinoma of the distal esophagus and the gastro-esophageal junction. Eur J Cardiothorac Surg.

[B18] Medical Research Council Oesophageal Cancer Working Group (2002). Surgical resection with or without preoperative chemotherapy in esophageal cancer. A randomized controlled trial. Lancet.

[B19] Samel S, Hofheinz R, Hundt A, Sturm J, Knoll MR, Wenz F, Queißer W, Post S (2001). Neoadjuvant radio-chemotherapy of adenocarcinoma of the oesophagogastric junction. Onkologie.

